# Tilting Together: An Information-Theoretic Characterization of Behavioral Roles in Rhythmic Dyadic Interaction

**DOI:** 10.3389/fnhum.2020.00185

**Published:** 2020-05-25

**Authors:** Dari Trendafilov, Gerd Schmitz, Tong-Hun Hwang, Alfred O. Effenberg, Daniel Polani

**Affiliations:** ^1^Institute of Pervasive Computing, Johannes Kepler University, Linz, Austria; ^2^Department of Humanities, Institute of Sports Science, Leibniz University Hannover, Hanover, Germany; ^3^Adaptive Systems, University of Hertfordshire, Hatfield, United Kingdom

**Keywords:** sensorimotor contingencies, interpersonal coordination, collaborative interaction, transfer entropy, information theory, causality, social interaction

## Abstract

Every joint collaborative physical activity performed by a group of people, e.g., carrying a table, typically leads to the emergence of spatiotemporal coordination of individual motor behavior. Such interpersonal coordination can arise solely based on the observation of the partners' and/or the object's movements, without the presence of verbal communication. In this paper, we investigate how the social coupling between two individuals in a collaborative task translates into measured objective and subjective performance indicators recorded in two different studies. We analyse the trends in the dyadic interrelationship based on the information-theoretic measure of transfer entropy and identify emerging leader-follower roles. In our experimental paradigm, the actions of the pair of subjects are continuously and seamlessly fused, resulting in a joint control of an object simulated on a tablet computer. Subjects need to synchronize their movements with a 90° phase difference in order to keep the object (a ball) rotating precisely on a predefined circular or elliptic trajectory on a tablet device. Results demonstrate how the identification of causal dependencies in this social interaction task could reveal specific trends in human behavior and provide insights into the emergence of social sensorimotor contingencies.

## 1. Introduction

In everyday joint physical activities humans often coordinate their motor behavior. Such interpersonal coordination emerges when two people dance, row a canoe, or carry an object together. In some cases, coordination of this kind could be controlled through a direct physical contact (e.g., dance), and in other cases it could be mediated by a rigid object (e.g., a table), or it can also be distantly coordinated without any physical contact. In such various types of social interaction, visual contact has different levels of importance, as humans typically coordinate their movements by detecting visual movement information (Schmidt et al., 1990), and this could lead to coordination even when it is not necessary for completing the task (Schmidt and O'Brien, 1997; Richardson et al., 2005). From a dynamical systems perspective, such visually mediated interpersonal coordination can be understood as a self-organized entrainment process of biological rhythms (Newtson et al., 1987; Schmidt et al., 1990).

Interpersonal coordination can be influenced by different modes of non-verbal communication (e.g., mimicry, gestures, and facial expressions) as a basis of social interaction (Vicaria and Dickens, 2016). Such non-verbal expressions could induce spatiotemporal coordination and could facilitate social entrainment between two or more individuals (Phillips-Silver and Keller, 2012). Non-verbal means of communication are generally faster than verbal in sharing action plans and strategies, when instant reaction is required in a joint task (Knoblich and Jordan, 2003). Non-verbal communication modes, supporting emergent coordination, stretch across a broad spectrum of perceptual modalities, like visual, kinesthetic, tactile, or auditory (Marsh et al., 2009). Dancers coordinate non-verbally during performance relying on visual as well as auditory cues (Waterhouse et al., 2014). Demos et al. (2012) reasoned that the spontaneous coordination would result from emergent perceptuo-motor couplings in the brain (Kelso, 1995). Keller suggested that online perceptual information might enhance the anticipation of one's own action, as well as the co-performer's action, in terms of developing common predictive internal models (Keller and Appel, 2010; Keller, 2012).

One limitation of interpersonal coordination research stems from the fact that studies (e.g., Schmidt and Turvey, 1994) usually require individuals to focus their visual attention directly toward the movements of their co-actor. The current study tested the coupling strength and the stability of interpersonal coordination in a task that required visual control of a ball on a tablet screen. The movement of the ball resulted from the joint action of both persons. Neither the effect from the own action nor the partner's action could be perceived in isolation. When agents engage in social interaction, a rich spectrum of possibilities arises: under some conditions, they act together as one single entity, in other conditions they may act as independent individuals. There is an interplay between intrinsic, cognitively driven coordination and coordination driven by the environment. The intrinsic coordination between the actions of interacting agents is a candidate for a measure of individuality or autonomy with respect to other agents (Bertschinger et al., 2008). In a cooperative task, when two agents use independent controllers under information processing constraints, they arrive at intrinsic coordination in order to overcome limitations of their environment (Harder et al., 2010).

Interpersonal synergies are higher-order control systems formed by coupling movement of two (or more) actors. Many different approaches have been utilized for the characterization of social couplings, such as autocorrelation, cross-correlation (Box and Jenkins, 1970), transfer entropy (Barnett et al., 2009), Granger causality (Granger, 1969), and their potential has been demonstrated in many applications (e.g., Valdes-Sosa et al., 2005; Arnold et al., 2007; Ryali et al., 2011). Interactive alignment was used to investigate interpersonal synergies in conversational dialog (Fusaroli et al., 2014; Fusaroli and Tylén, 2015). A key challenge is to design a suitable procedure that allows synchrony and turn-taking to spontaneously take place. Traditional interactive paradigms mainly consist of non-contingent social stimuli that do not allow true social interaction (Redcay et al., 2010). However, apparent interpersonal coordination could be merely incidental rather than reflecting true coordination—people may appear to coordinate their movements because they simultaneously execute similar motor programs, mediated by shared motor representations (Garrod and Pickering, 2004, 2009; Sebanz et al., 2006). In this study we addressed that by designing a performance oriented closed-loop interaction paradigm, which requires tightly-coupled motor coordination. A study, based on the perceptual crossing paradigm, investigates the direction of influence using discretized turn-taking events (Kojima et al., 2017).

Dynamical processes modeling the stable modes of intentional inter-limb coordination within (Haken et al., 1985) and between (Schmidt et al., 1998) individuals, can be represented by coupled oscillators. One of the main principled treatments of mutual synchronization in a network of oscillators was proposed by Kuramoto (1984) and is related to work of Bottani (1996), Pikovsky et al. (2001), Strogatz (2003), and Winfree (1967, 1980). Kuramoto (1984) developed a tractable mean-field model of coupled biological oscillators (Winfree, 1967), such as groups of chorusing crickets (Walker, 1969), flashing fireflies (Buck, 1988), or cardiac pacemaker cells (Peskin, 1975), which exhibits a spontaneous transition from incoherence to collective synchronization as the coupling strength is increased past a certain threshold. However, the original model relates to sinusoidal all-to-all couplings, which are not typical for biological systems. Strogatz (2003) introduced a not pure sinusoidal generalization, which also enables the addition of noise by a flux term. In the case of identical oscillators, perfect synchrony extends to time-delayed interactions, and when the oscillators are completely disorganized, different synchronized states can coexist with a stable incoherent state (Adlakha et al., 2012). Hanson's model of firefly entrainment, captured by an extension of the Haken-Kelso-Bunz equation (Kelso et al., 1990), specifies the eigenfrequency difference or frequency detuning between two rhythmic units. It reveals that human interpersonal rhythmic coordination is subject to the same dynamical laws as seen elsewhere in nature. Entrainment of unpredictable and chaotic systems was studied more recently by Dotov and Froese (2018).

New approaches from social neuroscience use imaging techniques, such as FMRI, fNIRS, and M/EEG, to study brain mechanisms in social interactions. One promising approach is hyperscanning, in which the brain dynamics of multiple subjects are studied simultaneously (Czeszumski et al., 2020). With EEG-hyperscanning, Sänger et al. (2012) found increased phase locking and phase coherence connection strengths in phases characterized by high demands on (musical) action coordination. Furthermore, oscillatory couplings between musicians' brains enabled the inference of leader-follower roles (Sänger et al., 2013). Similar observations were made by Dumas et al. (2010) in an imitation task, i.e., neuronal synchronization becomes asymmetric when one person is a leader and the other imitator. Konvalinka et al. (2014) demonstrated that multivariate decoding of inter-brain activity in an interactive task can identify the spontaneous emergence of leader-follower relationships within a dyad. Stephens and Galloway (2017) applied a quantitative information-theoretic approach for modeling the information exchange in healthcare teams in interactive navigation by transforming EEG-data into a stream of Shannon-entropy units characterizing team members' relationships.

Studies focusing on hyperscanning analysis of information flows between human brains require estimating the causal links between brains. Such causal links are established typically using Granger Causality or its frequency domain equivalent Partial Directed Coherence (PDC) (e.g., Astolfi et al., 2011, 2012). Previous results reveal stronger causal links during increased cooperative behavior and altruistic behaviors in decision-making tasks (Fallani et al., 2010; Ciaramidaro et al., 2018). Schippers et al. (2010) studied causal links in gesture communication using fMRI and Pan et al. (2017) using fNIRS between brains of cooperating lovers. Yun et al. (2012) investigated a paradigm for identifying the behavioral and the neural correlates of implicit cooperative social interaction. Leong et al. (2017) demonstrated that adults and infants show significant mutual neural coupling during social interactions. Liu et al. (2016) proposed a novel method for studying social cognition in the cooperative and obstructive game of Jenga. Naeem et al. (2012) explored mutual information on EEG data in social interaction tasks. Lobier et al. (2013) found that Phase Transfer Entropy detects the strength and direction of connectivity in the presence of noise characteristic for EEG data. The growing variety of hyperscanning analysis techniques suggest their exploratory nature and often the advantages and disadvantages of a specific method are not obvious. A key open research question relates to the neural substrates enabling the information flow between brains. In this respect it is crucial to emphasize the difference between information flow and synchronized neural activity between brains due to identical sensory input.

Despite such significant insights into the neuronal mechanisms of social interactions and social roles, Liu et al. (2018) pointed out that their behavioral correlates are still largely unclear and further research is needed to decompose the complicated mental constituent into basic psychological processes. The reciprocal influence in social interactions represents a major challenge with regard to the design of experiments. This is a starting point for the present study, which introduces a behavioral approach to quantify and investigate reciprocal influences and social roles. In a two-person cooperative tapping behavioral study using transfer entropy Takamizawa and Kawasaki (2019) identified leader/follower relationships which were consistent with subjective experiences.

## 2. Measures of Causal Relationship

Various measures of causal relationship exist, the main groups being model-based [e.g., Granger causality (Granger, 1969) or dynamic causal modeling (Friston et al., 2003)] or non-parametric methods [e.g., transfer entropy (Schreiber, 2000) or directed information (Massey, 1990)]. Granger causality is particularly useful when the interaction between the agents can be approximated well linearly and data has relatively low levels of noise (Nalatore et al., 2007).

Shannon mutual information, in conjunction with signal independent component analysis provides new aspects of brain-to-brain coupling in dyadic social interactions (Naeem et al., 2012), and reveals how the dynamic interaction unfold, determined by its specific properties. In the context of information theory, the key measure of information of a random variable is its Shannon entropy (Shannon, 1948). The entropy quantifies the reduction of uncertainty obtained when one actually measures the value of the variable. Therefore, if prediction enhancement can be associated to uncertainty reduction, it is expected that a causality measure would be naturally expressible in terms of information-theoretic concepts. Attempts to obtain model-free measures of the relationship between two random variables based on mutual information (MI) do not rely on any specific model of the data. However, MI says little about causal relationships, because of its lack of directional and dynamical information. Since MI is symmetric under the exchange of signals, it cannot distinguish driver and response systems, and furthermore, standard MI only captures the amount of information shared by two signals. In contrast, a causal dependence is related to the information being exchanged, rather than shared. The principle of maximum causal entropy provides causal analysis of the behavior of interacting systems, reflecting the causal dependencies between the processes (Ziebart, 2013; Ziebart et al., 2013). Building upon Massey's directed information (Massey, 1990) it extends random field models to settings with feedback, by providing a framework for estimating an unknown process based on its interactions with a known process.

Another information-theoretic framework, called transfer entropy, was proposed by Schreiber (2000) as a rigorous derivation of a Wiener causal measure. Assuming that two time series of interest *X* = *x*_*t*_ and *Y* = *y*_*t*_ can be approximated by Markov processes, transfer entropy computes the deviation from the following generalized Markov condition

(1) $$\begin{array}{l} p\left(y_{t+1}|y_{t}^{n},x_{t}^{m}\right)=p\left(y_{t+1}|y_{t}^{n}\right), \end{array}$$

where $x_{t}^{m}=\left(x_{t},\ldots ,x_{t-m+1}\right),y_{t}^{n}=\left(y_{t},\ldots ,y_{t-n+1}\right)$, and *m* and *n* are the orders (memory) of the Markov processes *X* and *Y*, respectively. Using the expected Kullback-Leibler divergence between the two probability distributions at each side of Equation 1, defines transfer entropy from *X* to *Y* as

(2) $$\begin{array}{l} TE\left(X\to Y\right)=\underset{y_{t+1},y_{t}^{n},x_{t}^{m}}{\sum }p\left(y_{t+1},y_{t}^{n},x_{t}^{m}\right)\log\frac{p\left(y_{t+1}|y_{t}^{n},x_{t}^{m}\right)}{p\left(y_{t+1}|y_{t}^{n}\right)}. \end{array}$$

Transfer entropy naturally incorporates directional and dynamical information, because it is inherently asymmetric and based on transition probabilities. Earlier efforts to understand causal relationships mostly relied on model-based approaches, such as Granger causality or dynamic causal modeling. In contrast, transfer entropy (TE) does not require a model of the interaction and is inherently non-linear. Thus, the sensitivity of transfer entropy to all order correlations becomes an advantage for exploratory analyses over Granger causality or other model-based approaches. This is particularly relevant when the detection of unknown non-linear interactions is required. Transfer entropy has seen a dramatic surge of interest in neuroscience recently, where it is used to estimate the information transfer between two tightly coupled processes. It requires the observation of multiple realizations of the processes, in order to estimate the associated probability density functions, provided stationarity assumptions. In this study, we investigated the applicability of TE as a measure characterizing causal dependence based on behavioral data in a simple collaborative motor task and demonstrated the relation of TE to standard performance metrics.

## 3. The Study

**Figure 1:**
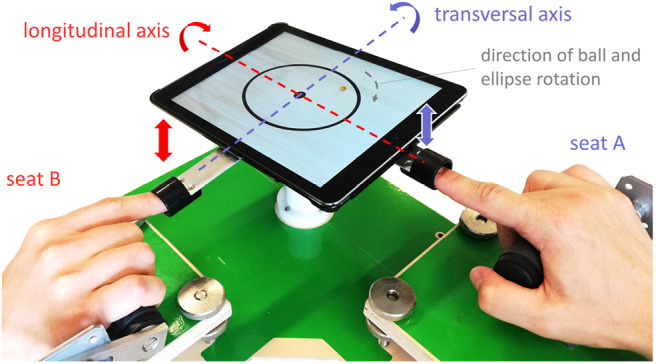
Experimental apparatus. Two participants moved a virtual ball on a circular or elliptic target line. A universal joint underneath the tablet limited the motion space to motions around the longitudinal and the transversal axes. Each participant controlled one tablet axis with upward and downward movements of his index finger tip. Therefore, the task required the coordination of the individual actions.

The current study builds on the tetherball paradigm introduced in Hwang et al. (2018), and is implemented on a tablet computer (see Figure 1). With rhythmic finger movements, a pair of participants had to tilt the tablet in order to rotate a ball along a predefined circular target trajectory (experiment A). One measure of joint task performance in this scenario is the average target tracking precision, i.e., the spatial error between the ball and the target trajectory. Since Hwang et al. (2018) reported for the circular task that the error reaches a plateau after a few trials, we applied an alternative task in experiment B, where the participants had to track a rotating ellipse instead. In each condition, we evaluated the tracking accuracy as a measure of task performance as well as the information flow (i.e., transfer entropy) as a measure of mutual influence between the two participants based on their actions. Participants were also asked to report on their subjective experience of interpersonal coordination. Our aim was to gain an initial insight into the utility of information-theoretic functionals, such as transfer entropy and its variants, for the characterization of social couplings based on behavioral data. We investigated the following research questions:

- (RQ1) What is the relation between measured interpersonal coordination and achieved task performance?
- (RQ2) Is there a correlation between objective and subjective measures of interpersonal coordination?
- (RQ3) Can transfer entropy provide insights into specific behavioral patterns and identify distinct roles within a dyad?

## 4. Materials and Methods

### 4.1. Participants

We collected data from 76 participants (provided in the Supplementary Material) and reanalyzed data of the 72 participants from the study of Hwang et al. (2018). In total, the data from 46 females and 102 males were analyzed (mean age: 25.7 years, *SD*: 4.6 years). All participants reported to healthy, and none of them had overt psychic or cognitive impairments. They were tested for normal eyesight with the Landolt rings chart (Jochen Meyer-Hilberg) and for normal hearing abilities with the HTTS audio test (SAX GmbH). All participants gave their written informed consent to the study. The study protocol and all documents had been independently reviewed and pre-approved by the Ethics Committee of the Leibniz University Hannover.

### 4.2. Experimental Apparatus

The participants sat in front of the experimental apparatus, which is shown in Figure 1. With their dominant hand, they grabbed an adjustable handle and inserted the tip of their index finger into a lever, which was attached to a tablet (iPad Air, Apple Inc.). A universal joint underneath the tablet allowed to move the tablet around its transversal and longitudinal axes as indicated by the dashed lines in Figure 1. Rotations along the vertical axis were not possible. With upward and downward movements of the index finger, each participant controlled one dimension; i.e., participant A on seat A controlled the transversal axis and participant B on seat B controlled the longitudinal axis. The screen (1,024 * 768 pixel, 60 Hz) displayed a target line and a virtual ball. The target line had a width of 0.29 cm and had either the shape of a circle (diameter: 8.95 cm, experiment A) or the shape of an ellipse (axes length: 10.94 and 8.47 cm, experiment B). The ellipse rotated with 2.5 revolutions per minute. The ball is illustrated as gray dot in Figure 1. It had a diameter of 0.58 cm and was connected by an invisible elastic spring to an anchor at the center of the screen. The spring force was just strong enough to pull the ball to the anchor, when the tablet was in a horizontal position.

### 4.3. Paradigm

By tilting the tablet, the participants could move the ball around the central anchor. The instruction was to move the ball clockwise and as accurately as possible on the target line. This was only possible if both players contributed to the task and tilted the tablet around both axes in a certain pattern and with a certain amplitude of frequency. Since both players sat in an angle of 90° to each other, optimal performance was achieved by synchronizing finger movements with a 90° phase-difference [see Video 1 in the Supplementary Material of the earlier publication (Hwang et al., 2018)].

Two participants of the same gender performed as dyad. Twenty-eight female and 22 male dyads performed the task with a circle as target line (experiment A) and 24 dyads performed the task with an ellipse as target line (experiment B). Data from 36 dyads from experiment A had been published before in Hwang et al. (2018). These authors focused on a different topic compared to the present study by investigating the impact of different types of auditory feedback on joint performance in the tablet task. Thereto, they compared the performance between four groups, which played under different perceptual conditions. One group received purely visual information and three groups visual plus auditory information. The auditory information either provided knowledge of performance by transforming the angular velocity measured by the tablet's gyroscopes into sound (broom sweeping sound) or knowledge of results by transforming the two-dimensional ball position on the screen sound (synthesized violin, details of the parameter-sound-transformation are described in Hwang et al., 2018). Yet unpublished data from further 14 dyads with a different tilt sound (synthesized violin) but similar performance compared to the participants from Hwang et al. (2018) were also included in the analyses. We believe that reanalyzing these data sets is reasonable considering the different study goals and types of analyses: Whereas Hwang et al. (2018) focused on comparisons between groups, the present study focused on the intra-dyadic coupling between two players and leader-follower relationships. An influence of sound condition on leader-follower-relationships was not expected, because both players of one dyad had the same perceptual condition. Thus, we combined the data of all groups for the analyses of the present study. Nevertheless, we analyzed whether sound condition influences social coupling by comparing the transfer entropy measures between groups. The participants from experiment B were not provided with artificial auditory information. This paradigm was specifically designed to investigate leader-follower relationships, for which we explored transfer entropy as an objective measure of causal dependence. This could serve as an initial step toward the characterization of more general social sensorimotor contingencies.

### 4.4. Procedure

The participants familiarized with the experimental apparatus in a 2-min practice phase. During that, each participant controlled his own ball and tried to track a target zone (diameter 0.58 cm), which moved randomly along the longitudinal axis for participant A and along the transversal axis for participant B. The main task was divided up into 1-min trials. Because the task was attentionally demanding, a 2-min break was introduced after every five trials. During that break, the participants were allowed to talk with each other, but instructed not to talk about the experiment. The participants from experiment A performed fifteen trials. In experiment B, the participants performed thirty trials. In the latter group, the participants exchanged their seats after every five trials; i.e., each participant performed fifteen trials in seat A and fifteen trials in seat B. This procedure was chosen, because the 90° angles of the seating positions and the resulting 90° phase-difference of the player's actions might influence the leader-follower relationship: The ball first passes player A, then player B; therefore, player A might take the leadership role more likely as player B. By exchanging seating positions, we could analyze leader follower relationships independently from this effect. To assess subjective experiences, the participants were interviewed at the end of the experiment using standardized questions. In an open question, the participants were asked to describe who they felt was leading the interaction, if any at all. Furthermore, participants had to rate on a 7-point Likert scale how much they felt they helped their partner (Q1) and how much they felt their partners helped them (Q2).

### 4.5. Dependent Measures

The tablet recorded the path of the ball (from the visual display) and the angular velocity (from the built-in gyroscope sensor) at the sampling rate of 60 Hz. We measured task-related performance based on the absolute error between the target trajectory and the actual ball trajectory. Furthermore, we computed the transfer entropy between the two players' actions per trial, using the Kraskov-Stogbauer-Grassberger transfer entropy estimator (Kraskov et al., 2004) from the JIDT toolkit (Lizier, 2014), based on the raw tablet gyroscope time series for the transversal and longitudinal axes, which correspond to the finger movements of players A and B, respectively. We computed the transfer entropy and the mean levels of the absolute error over each 1-min trial while discarding the initial 8.3 s (500 samples at 60 Hz) in every trial for initialization reasons.

### 4.6. Data Reduction

In order to relate the user ratings of perceived collaboration to the objective levels of transfer entropy we had to perform specific conversion on the subjective data. We transcribed the informal verbal answers from both players with discrete numerical representations {1, 0, −1}, meaning, respectively {I was leader, there was no leader at all, partner was leader}. Using this numerical representation, we subtracted the values in order to compute the difference in the opinions and took the sign of the result. Furthermore, for the closed questions, we calculated the difference between the ratings of the partners and took the sign of the result.

## 5. Results

**Figure 2:**
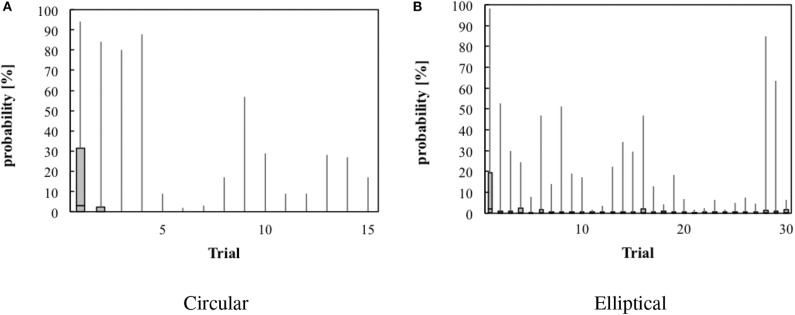
Box and whisker plots illustrating the probability that the transfer entropy measures are random. Data of experiment A (circular target) are shown in **(A)**, data of experiment B in **(B)**. **(A)** Circular. **(B)** Elliptical.

In order to validate the transfer entropy measure for this particular data set, we performed surrogate data testing with 1,000 random pairings for each TE value in each trial. The results of the surrogate data testing are shown in Figure 2. Using Wilcoxon tests on the surrogate data, we compared the outcome of each trial against the constant 0.05, which corresponds to the conventional significance level. The TE values were significant in all trials of experiment A (Figure 2A) (at least *p* < 0.05) and in most trials of experiment B (Figure 2B, *p* >0.05 in trials 1, 4, 6, and 28, at least *p* < 0.05 in all other trials; results were Bonferroni-Holm-corrected). Transfer entropy estimates typically stabilized at 1,000 samples, with some variability across trials and subjects. However, such trends depend on sampling rates and on the performance of subjects. For our analysis, we computed TE on a trial base, i.e., ca. 3,000 data points.

### 5.1. Learning Effect

**Figure 3:**
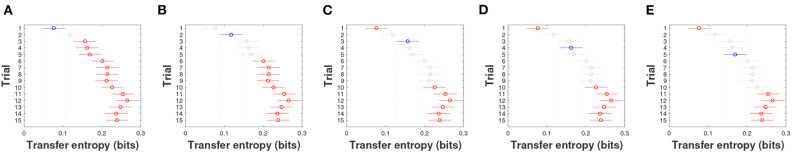
Mean total transfer entropy levels (*TE*(*A* → *B*) + *TE*(*B* → *A*)) per trial averaged over all 50 pairs in Experiment A, revealing the learning effect and the statistical significant differences of the transfer entropy means between trials one to five in blue and the trials in red (in separate subplots). The differences were not significant for the rest of the trials. **(A)** Trial 1 vs. rest. **(B)** Trial 2 vs. rest. **(C)** Trial 3 vs. rest. **(D)** Trial 4 vs. rest. **(E)** Trial 5 vs. rest.

**Figure 4:**
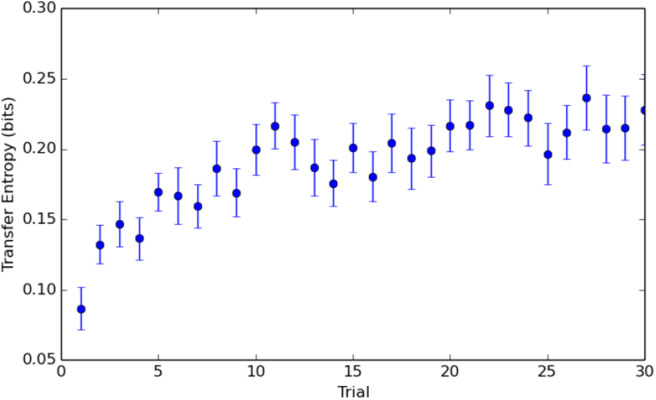
Mean total transfer entropy levels (*TE*(*A* → *B*) + *TE*(*B* → *A*)) per trial averaged over all 24 pairs in Experiment B, revealing the learning effect.

The trend in mean total transfer entropy levels averaged over all pairs, reveals a pronounced learning effect during both experiments (see Figures 3, 4). Accordingly, trials were significantly different (experiment A: *F*(14, 630) = 38.81, *p* < 0.001; Experiment B: *F*(29, 667) = 5, 09, *p* < 0.001). With the circular target, the trend is more consistent and with lower variance than with the elliptic target, presumably due to repeated seat exchanges in the latter. Figure 3 reveals the statistical significant differences of transfer entropy means between trials one to five in blue and the trials in red in Experiment A, computed with Tukey-Kramer (HSD) multiple comparison test. The differences were not significant for the rest of the trials. These figures suggest that Experiment A and Experiment B, although slightly different by design, are TE-invariant and reach plateau at ~0.25 bits.

Neither in Experiment A nor in Experiment B, seat position had a significant effect on the size of transfer entropy. Within experiment A, there was a significant difference between groups with different auditory conditions [*F*_(4, 45)_ = 2.85, *p* = 0.035]: On average, transfer-entropy was higher for the participants that heard a broom sweeping sound (0.24, *SD*: 0.07 bits) compared to participants that heard a synthesized violin sound of the tablet tilt velocity (0.16, *SD*: 0.05 bits, *p* = 0.024).

### 5.2. Social Roles and TE Relevance

**Figure 5:**
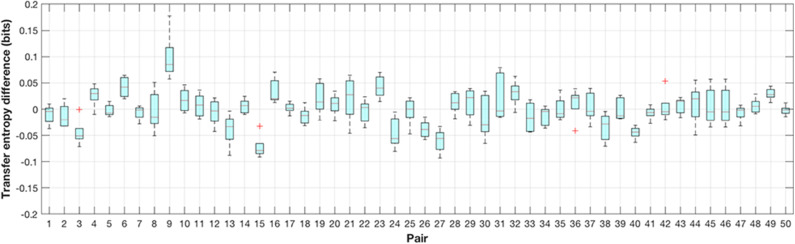
Distribution of transfer entropy differences (*TE*(*A* → *B*) − *TE*(*B* → *A*)) computed on each 1 min trial for all pairs in experiment A (circular target). Consistent positive levels reveal higher information transfer from player A to player B and vice versa, negative levels—from player B to player A, which suggests specific social dynamics (e.g., pairs 3, 4, 6, 9, 15, 16, 23, 24, 26, 27, 38, 40, 49). Data for pairs from 1 to 36 is from an earlier study (Hwang et al., 2018).

In order to get further insight into the interpersonal dynamics of the emerging social interaction, we computed the differences in transfer entropy (*TE*(*A* → *B*) − *TE*(*B* → *A*) and *TE*(*B* → *A*) − *TE*(*A* → *B*)) between both players for each 1 min trial while taking into consideration the actual seating. The distribution of results on a trial level for all pairs from Experiment A suggests potential leader-follower roles for particular pairs despite the high variability (e.g., pairs 3, 4, 6, 9, 15, 16, 23, 24, 26, 27, 38, 40, 49) (see Figure 5). Predominantly positive values reflect consistently higher information transfer from player A to player B and vice versa, negative levels—from player B to player A, which provides a base for making specific inferences about leader-follower relationships spontaneously emerging during the non-verbal social interaction.

**Figure 6:**
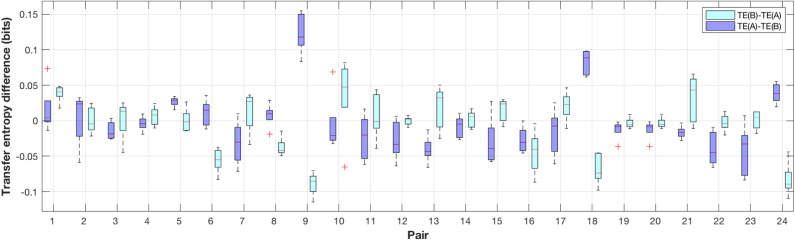
Distribution of transfer entropy differences (*TE*(*A* → *B*) − *TE*(*B* → *A*) and *TE*(*B* → *A*) − *TE*(*A* → *B*)) on a trial level for all pairs in experiment B (elliptical target). The results are split into two subsets per pair (blue/cyan), corresponding to consistent seating of players over different trials. Pairs 8, 9, 10, 18, and 24 exhibit opposite trends in the two seating arrangements, suggesting that their social roles were not affected by the particular seating.

In order to compensate for the alternating seating arrangements in experiment B, we split the results into two subsets per pair (shown in blue and cyan in Figure 6), corresponding to the consistent seating of both players during the experiment. Blue color denotes trials in which player A sat in seat A and cyan in which player A sat in seat B. The figure reveals how repetitive seat exchanges affect the coordination trends. For example, for pair 9, the results show that player A transfers more entropy than player B regardless of the seat, reflected by positive and negative transfer entropy differences in the two different seating arrangements, which suggests consistent roles for this pair throughout the experiment. Similar trends are visible also for pairs 8, 10, 18, and 24, suggesting that their social roles were not affected by the particular seating. Identifying such coherent patterns of social behavior was one of the main goals of this study and in the next paragraph we demonstrate how these objective measures correspond to the subjective levels of interpersonal coordination measured by user experience questionnaires.

**Figure 7:**
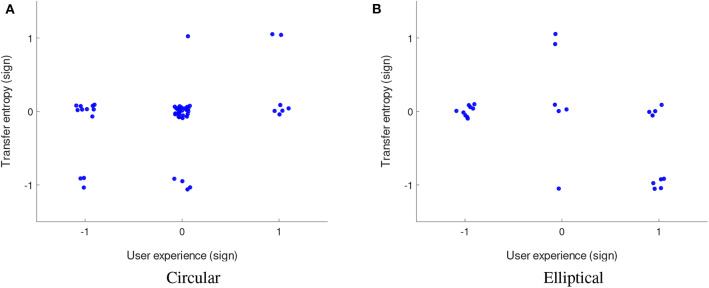
Significant Pearson correlation between the sign functions of the transfer entropy difference and user experience difference for the open ended question in experiment A (circular target, **A**) (Rho = 0.34, *p* <0.02) and for Q2 in experiment B (elliptical target, **B**) (Rho = −0.44, *p* <0.04). To avoid overlapping points and improve visibility, the values (−1, 0, 1) of the sign functions are perturbed with white noise.

The participants rated both the support for (Q1) and from their partners (Q2) as high [Experiment A: medians = 6, interquartile ranges (IQRs) = 1; experiment B: medians = 5, IQR = 1 (Q1) and 2 (Q2)]. Furthermore, 18 percent (Experiment A) and 19 percent (Experiment B) of the participants answered that they were the leader, while 9 percent (Experiment A) and 17 percent (Experiment B) saw their partner as leader. Neither in Experiment A nor in Experiment B did these variables differ significantly between both players. There was no significant correlation between the open ended questions and Q1 or Q2 in either of the experiments. However, we found a significant correlation (Rho = 0.42, *p* < 0.00002, 95% CI [0.24, 0.57]) between Q1 and Q2 in Experiment A. Since in both studies subjective user experience was collected via questionnaires only at the end of the experiment, we were not able to correlate the user ratings with transfer entropy levels on a trial base. Instead, we took the last third of the trials for each pair and considered the reduced subsets representative for the subjective ratings provided at the end. This assumption essentially takes into account both the learning and the memory effect. Furthermore, since the main goal of this study was to identify specific leader-follower relationships based on the direction of influence between the two players and not on the exact values, which are only important for providing the order of magnitude, we took the sign functions of both the transfer entropy differences and the user ratings differences. Following this approach, we computed the Pearson correlation between the transfer entropy differences of the last third of the trials and the user experience differences using their sign functions and found a significant correlation (Rho = 0.34, *p* < 0.02, 95% CI [0.07, 0.57]) for the open ended question in experiment A (see Figure 7A). Furthermore, we found a significant correlation (Rho = −0.44, *p* < 0.04, 95% CI [−0.7, −0.04]) for Q2 in experiment B (see Figure 7B). The negative correlation resonates well with the content of Q2, as Q1 and Q2 have opposite meanings in inferring a potential leadership. Here, we assume that stronger sense of leadership is associated with higher ratings of one own's influence (or help) and lower ratings of the other's influence (or help).

### 5.3. Leader-Follower vs. Performance Trends

**Figure 8:**
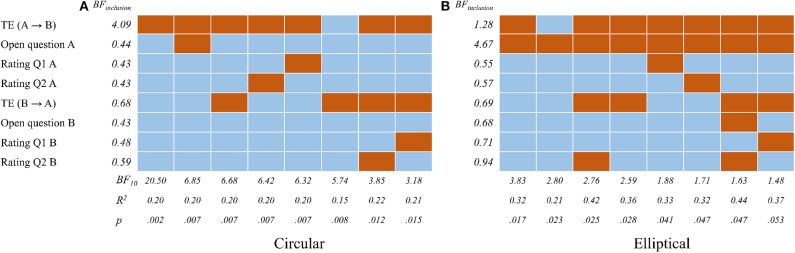
Bayesian linear regression analyses with performance error as criterion variable and *TE*(*A* → *B*), *TE*(*B* → *A*) and subjective measures (open question, Q1 and Q2) from participants A and B as predictor variables. The Bayes factor BF10 describes how likely the data occur under a regression model with the highlighted predictors of one row; the inclusion Bayes factor (BFinclusion) describes how likely the data occur under models that include the respective predictor. **(A)** Circular. **(B)** Elliptical.

To analyze the relationship between achieved task performance and objective as well as subjective measures of coordination, we first performed Bayesian linear regression analyses. Values deviating more than two standard deviations from the group mean were excluded from the analyses. As task performance is represented by the mean error, it was chosen as criterion variable. Predictors were *TE*(*A* → *B*), *TE*(*B* → *A*) and the subjective data from the questionnaires. In experiment A, the Bayes factor (*BF*10 = 20.50, percentage error < 0.001) was largest for a model including *TE*(*A* → *B*) as predictor [*R*2 = 0.20, *F*_(1, 45)_ = 11.20, *p* = 0.002]; i.e., the data were 20.5 times more likely under this model than under the null model. In experiment B, the largest Bays factor was achieved for a model with the predictors *TE*(*A* → *B*) and the coded answer from participant A to the open question [*BF*10 = 3.83, percentage error < 0.01; *R*2 = 3.83, *F*_(2, 21)_ = 4.99, *p* = 0.017]. Figure 8 shows alternative models sorted by their Bayes factor. The analyses of both experiments suggest that among the tested variables measures from participant A are the most important predictors for the joint performance—despite the seat exchange in experiment B.

**Figure 9:**
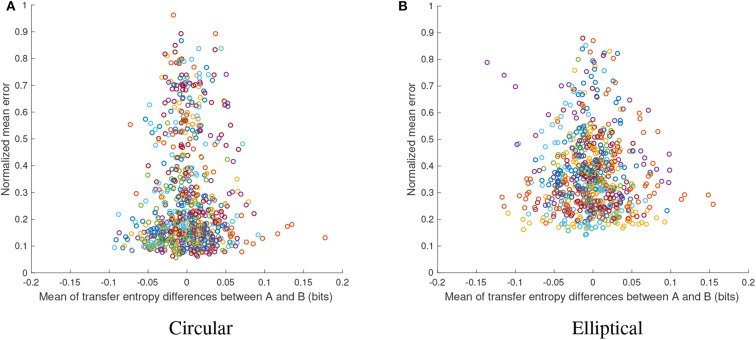
Performance (normalized mean absolute error) vs. transfer entropy differences per trial for all pairs from experiment A (**A**, circular target) and from experiment B (**B**, elliptical target). In both studies, the point densities have a characteristic bell shape, highlighting particular trade-offs.

Another interesting observation is the relation between the transfer entropy differences and the normalized mean absolute error, which is shown in Figure 9A for experiment A and in Figure 9B for experiment B. In both studies, the point densities have a characteristic bell-shaped form, reflecting that low performance is associated with low levels of transfer entropy differences. This suggests that in cases of (i) low *TE*(*A* → *B*) and low *TE*(*B* → *A*) or (ii) quasi equal transfer entropies (*TE*(*A* → *B*) − *TE*(*B* → *A*) ≈ 0), performance can be high or low alike, however, for more significant and disparate levels of transfer entropies performance is typically higher. This provides an interesting insight into the social aspect of the experimental paradigm, i.e., more pronounced behavioral roles of leader-follower eventually lead to higher performance, although such behavior is not necessary, as high performance could be achieved even with less-structured or more-balanced behavior from both partners. The results also suggest that the achieved performance per level of coordination was higher for the circular than for the elliptical target, revealed by lower absolute error yielded at similar levels of interpersonal coordination. This confirms, as expected, that the more difficult elliptic task requires higher degree of social coordination between participants.

## 6. Discussion

This study investigated the social dynamics of interpersonal coordination in two proximal collaborative experiments. In the tetherball paradigm, participant pairs were asked to tilt the tablet together for the task. We compared the mean levels of the error, transfer entropy and subjective ratings in our analysis. We hypothesized that stable rhythmic patterns of coordination would emerge in the course of interaction, which would be measurable with information-theoretic functionals. Our aim was to quantitatively identify and explain observed trends in the social aspect of interpersonal interaction. To test these predictions, we analyzed two types of studies, which examined the movement patterns of pairs of individuals performing a collaborative circular and elliptic motion jointly through the coordination of their movements. In both studies, the participants were only instructed to maximize their task performance, without explicitly guiding them to focus on their interpersonal entrainment and coordination. The topic of coordination was raised only in the user experience questionnaire, filled in after the experiment was completed. This ensured that the social dynamics, observed during the experiment, emerged spontaneously and not by instruction. The results presented in Figures 8, 9, provide the answer to RQ1, which is invariant for both studies. Correlations, provided in Figure 7 reflect RQ2, although the significance is rather sporadic and not across the board. Figures 5, 6 provide evidence supporting a positive answer to RQ3 in respect to both studies. The transfer entropy measure clearly emphasizes the learning effect, although the trend is not continuously monotonic. However, considering the fact that the relationship between task performance and interpersonal coordination (as measured by transfer entropy) is not monotonic, such a trend is plausible. The results show that tightly coupled interaction leading to higher coordination levels improve task performance, however it is not indispensable. Different, e.g., loosely coupled, social behavior might achieve a good performance as well. The effect of exchanging seats in the elliptic study introduced higher variability in the transfer entropy measure, although the main trends remained consistent.

In summary, the results of both experiments were consistent regarding the validation of TE as objective measure for interpersonal coordination in this task, as well as the significant correlations between TE and joint task performance, and with respect to the identification of leader-follower roles on a descriptive level. Similarly to Takamizawa and Kawasaki (2019), we hypothesized that TE from leader- to follower-like behavior was large and vice versa, from follower to leader—small. When the TE in both directions was on the same level—equally high or equally low—we hypothesized that there was no pronounced leader-follower relationship according to our measure. Different were the correlations between subjective and objective measures. Despite these differences, the results of both studies suggest that TE might be a useful tool for studying factors for subjective experiences in social interactions.

The seat exchange provided one important insight in this study, namely the seat-invariance of social roles within certain dyads, since regardless of the seat the flow of entropy kept the direction from one player to the other (Figure 9). Furthermore, the results of the Bayesian linear regression analyses (Figure 8), which allow to compare the predictive value of *TE*(*A* → *B*) and *TE*(*B* → *A*) on the joint task performance, indicate that social roles also depend on the first seating arrangement and preserve when the participants change their seats. This suggests the potential of the applied measure to infer simple social relationships based solely on behavioral data recorded in a smooth rhythmic repetitive interaction. It is well-known that information-theoretic functionals, such as transfer entropy, require large amounts of data in order to provide reliable estimates. Since trials were considerably short, the analysis seem to have been enhanced by the simplicity of interaction.

On the other hand, the simplicity of the experimental paradigm seems to have raised challenges in the subjective evaluation, particularly when addressing the sense of collaboration and interpersonal coordination. We used an explicit open-ended question for establishing the potential leadership within the pairs, as well as a few more subtle indirect questions rated on a Likert scale. The subjective data did not provide consistent trends, which suggests how difficult it was to subjectively evaluate one's own performance in the social aspect of such collaborative interaction. Another issue complicating the subjective data analysis was the fact that questions were answered only once at the end of the experiment and therefore the ratings did not usually apply for all trials (particularly not for the earlier ones). That may have been one potential reason for the participants' confusion in the evaluation, as it was left up to them to decide how to rate the whole experiment (including earlier and later trials alike), providing the otherwise complicated nature of this judgement. In order to make sense of the ratings, we applied a simple (non-distorting) linear transformations on the subjective data which kept the major trends, and correlated the results to the corresponding major trends in the objective measure of coordination (i.e., transfer entropy).

The results of this study suggest the potential of model-free measures of information transfer, such as the transfer entropy, for the analysis of the social aspects of highly interactive collaborative studies, particularly involving simplistic rhythmic controls. Other methods, such as lagged cross-correlation or Granger causality (Granger, 1969), require stationarity and normality assumptions or pre-defined models. Transfer entropy has seen a dramatic surge of interest in neuroscience recently (Wibral et al., 2014), where it is used to estimate the information transfer between two tightly coupled processes. We extend its field of application to less tightly coupled stochastic processes, which form a closed loop with hundreds of milliseconds of lag and involve the full human sensorimotor and decision making hierarchy of control.

The task required the participants to anticipate the combined effect of their joint actions. This might enhance participants' understanding of their own and their partner's actions as well as joint actions, which positively affects interpersonal coordination. In addition, previously published literature (Schmidt and Richardson, 2008; Keller et al., 2014; Lang et al., 2016; Loehr and Vesper, 2016) highlights the significance of rhythmical movement components in interpersonal coordination. Additionally, there is evidence that the rhythmic component during interpersonal coordination reduces practice effort and errors (Lang et al., 2016; Loehr and Vesper, 2016).

Overall, the results supported the hypothesis that this type of collaborative interaction intrinsically motivates the emergence of interpersonal rhythmic coordination, which could be objectively characterized with information-theoretic tools. Our analysis provides quantitative evidence for the emergence of leader-follower relationships, guided by the general principle of perceptual–motor coordination, and is an initial step toward defining more general social sensorimotor contingencies. This evidence was consistent across the experiments and was not diminished by task difficulty levels or seating arrangements of the participants. Similar leader-follower relationships have been identified in other studies (Konvalinka et al., 2014; Takamizawa and Kawasaki, 2019) using different social interaction tasks and/or different analytical methods. Takamizawa and Kawasaki (2019) apply transfer entropy on behavioral data as well, however their study is based on a discrete tapping task, while our study explores a highly interactive continuous paradigm. Our analysis suggests that although subjects tend to steadily improve their coordination skills over time and produce tightly coupled rhythmic control signals, they do not necessarily apply such techniques in order to improve performance.

Finally, the current study has important implications for future research on the social psychological aspects of interpersonal coordination as it reveals the potential of non-parametric information-theoretic methods for quantifying behavioral trends in joint cognitive systems, which are typically identified qualitatively by human observers. The tetherball paradigm provided an easy to learn natural test environment and a basis for examining the interpersonal processes involved in mutual entrainment.

## 7. Conclusion

The characterization of causal dependence can be approached with a variety of methods, and depending on the experimental paradigm in some scenarios certain methods might be more appropriate than others. In this study, we applied the information-theoretic measure of transfer entropy for quantifying the emergent social sensorimotor contingencies in the scope of two studies. While the results look promising, further work is required to explore the range of applicability of this approach for measuring interpersonal coordination in a variety of diverse tasks. Future studies need to address carefully the subjective aspect in the design process. It might be interesting to investigate paradigms in which task performance is inherently correlated with interpersonal coordination. An important aspect for future research is how motor learning and the emergence of interpersonal coordination are related to each other and how that can be expressed with objectives metrics. These aspects are closely related to the perception of kinematics—human control movements or reference object's movements (e.g., a table). Having an objective tool for inferring the level of causal dependence from behavioral data could improve current studies and could facilitate the identification of socializing sensorimotor contingencies in future research.

## Data Availability Statement

All datasets generated for this study are included in the article/Supplementary Material.

## Ethics Statement

The studies involving human participants were reviewed and approved by Ethics Committee of the Leibniz University Hannover. The patients/participants provided their written informed consent to participate in this study.

## Author Contributions

DT wrote the background and the main parts related to the information-theoretic analysis. GS wrote the experimental methodology part. T-HH together with AE and GS developed the paradigm and the experimental design. T-HH realized the software development. AE and GS supervised the data collection. The information-theoretic analysis and major part of the results were realized by DT and DP, supported by GS and T-HH. All authors critically revised the manuscript.

## Conflict of Interest

The authors declare that the research was conducted in the absence of any commercial or financial relationships that could be construed as a potential conflict of interest.
